# Correction to *Annals of the Child Neurology Society* articles

**DOI:** 10.1002/cns3.20097

**Published:** 2025-02-20

The below Conflicts of Interest related to membership on the ACNS editorial board were missing in the following articles.

van Haren KP, et al. Vitamin D status and latitude predict brain lesions in adrenoleukodystrophy. *Ann Child Neurol Soc*. 2023;1(2):155‐161. doi:10.1002/cns3.4

In the above article, the following sentence should have been included in the Conflicts of Interest section: “J. B. L. is a member of the ACNS editorial board.”

Wanigasinghe J, et al. Demographic characteristics and clinical presentation of infants with infantile epileptic spasms syndrome and their response to therapy: data from Sri Lanka Infantile Spasms Registry. *Ann Child Neurol Soc*. 2023;1(2):137‐143. doi:10.1002/cns3.20014

In the above article, the Conflicts of Interest section should have read, “Jithangi Wanigasinghe is a member of the ACNS editorial board. The remaining authors declare no conflicts of interest.”

Mohammadpour Touserkani F, Andriotis T, Zhang Y, Pavlakis S. Cerebral venous thrombosis and B12 deficiency. *Ann Child Neurol Soc*. 2023;1(2):152‐154. doi:10.1002/cns3.9

In the above article, the Conflicts of Interest section should have read, “Steven Pavlakis is a member of the ACNS editorial board. The remaining authors declare no conflicts of interest.”

Bansal S, et al. A question prompt list for sudden unexpected death in epilepsy. *Ann Child Neurol Soc*. 2023;1(2):144‐148. doi:10.1002/cns3.20027

In the above article, these two declarations should have been included in the Conflicts of Interest section: “Dr. Shellhaas is a member of the ACNS editorial board.” and “Dr. Lemmon is also a member of the ACNS editorial board.”

We apologize for this error.

